# Cystine reduces tight junction permeability and intestinal inflammation induced by oxidative stress in Caco-2 cells

**DOI:** 10.1007/s00726-021-03001-y

**Published:** 2021-05-15

**Authors:** Tatsuya Hasegawa, Ami Mizugaki, Yoshiko Inoue, Hiroyuki Kato, Hitoshi Murakami

**Affiliations:** Institute of Food Sciences and Technologies, Ajinomoto Co., Inc, Kanagawa, 210-8681 Japan

**Keywords:** Cysteine, Glutathione, Inflammation, Intestinal barrier, Oxidative stress, Claudin-4

## Abstract

Intestinal oxidative stress produces pro-inflammatory cytokines, which increase tight junction (TJ) permeability, leading to intestinal and systemic inflammation. Cystine (Cys2) is a substrate of glutathione (GSH) and inhibits inflammation, however, it is unclear whether Cys2 locally improves intestinal barrier dysfunction. Thus, we investigated the local effects of Cys2 on oxidative stress-induced TJ permeability and intestinal inflammatory responses. Caco-2 cells were cultured in a Cys2-supplemented medium for 24 h and then treated with H_2_O_2_ for 2 h. We assessed TJ permeability by measuring transepithelial electrical resistance and the paracellular flux of fluorescein isothiocyanate–dextran 4 kDa. We measured the concentration of Cys2 and GSH after Cys2 pretreatment. The mRNA expression of pro-inflammatory cytokines was assessed. In addition, the levels of TJ proteins were assessed by measuring the expression of TJ proteins in the whole cells and the ratio of TJ proteins in the detergent-insoluble fractions to soluble fractions (IS/S ratio). Cys2 treatment reduced H_2_O_2_-induced TJ permeability. Cys2 did not change the expression of TJ proteins in the whole cells, however, suppressed the IS/S ratio of claudin-4. Intercellular levels of Cys2 and GSH significantly increased in cells treated with Cys2. Cys2 treatment suppressed the mRNA expression of pro-inflammatory cytokines, and the mRNA levels were significantly correlated with TJ permeability. In conclusion, Cys2 treatment locally reduced oxidative stress-induced intestinal barrier dysfunction possively due to the mitigation of claudin-4 dislocalization. Furthermore, the effect of Cys2 on the improvement of intestinal barrier function is related to the local suppression of oxidative stress-induced pro-inflammatory responses.

## Introduction

The intestinal epithelium, composed of a single layer of cells, is a mucosal barrier that maintains the luminal environment of the intestine (Turner 2009) and regulates the permeation of luminal noxious substances, such as bacteria, toxins, and dietary antigens, into the intestinal cells (Claude and Goodenough 1973; Farquhar and Palade 1963; Powell 1981). Stressful stimuli, such as pregnancy, administration of drugs, and endurance exercise, induce intestinal barrier dysfunction, causing intestinal inflammation (Camilleri 2019). Chronic inflammation of the gastrointestinal tract leads to several serious intestinal diseases, such as inflammatory bowel disease (IBD) and colorectal cancer (CRC) (Michielan and D'Inca 2015; Terzic et al. 2010). In athletes, the intestinal barrier function is impaired locally due to exercise stress and secondarily due to systemic inflammation (Lambert 2009). Strenuous exercise induces heat-stress and oxidative stress in intestinal epithelial cells, injuring the intestinal barrier. Strenuous exercise also causes systemic inflammation, such as a rise in circulating concentrations of interleukin (IL)-6 released from muscle during exercise (Hennigar et al. 2017), which is likely associated with intestinal barrier dysfunction. Intestinal inflammation elevates the risk of mood disturbance, fatigue, and depression (Clark and Mach 2016). Therefore, there is an urgent need for an effective approach to improve and protect the intestinal barrier function.

Reactive oxygen species (ROS) are produced by various stresses such as exercise-induced ischemia, high-fat and high-carbohydrate diet-induced nutritional stress, and injuries due to ischemia/reperfusion (Bhattacharyya et al. 2014; Di Dalmazi et al. 2016; Rani et al. 2016; Rincon-Cervera et al. 2016; van Wijck et al. 2011). ROS, such as hydrogen peroxide and nitric oxide, disrupt tight junctions (TJs) and elevate paracellular permeability in vitro (Han et al. 2004; Rao et al. 2000). TJ proteins, such as zonula occludens-1 (ZO-1), occludin, and claudin-1 and -4, regulate the paracellular transport of ions, molecules, and water (Suzuki 2013). ZO-1 and cytosolic scaffold proteins form the intracellular domain of TJ and maintain the TJ structure. Occludin, claudin-1 and -4 create a barrier against small ions and macromolecules. The increase in TJ permeability leads to the translocation of lipopolysaccharide (LPS) into the blood circulation, triggering the release of pro-inflammatory cytokines such as tumor necrosis factor-α (TNF-α), IL-1β, or IL-6 (Raetz and Whitfield 2002). These cytokines activate NF-κB signaling pathways in epithelial cells, decreasing the expression of TJ proteins, such as ZO-1 and occludin, and increasing TJ permeability (Al-Sadi and Ma 2007; Ma et al. 2004). Other studies have reported that oxidative stress does not change the expression levels of TJ proteins in whole cells but changes the localization of TJ proteins from the apical membrane to the lateral membrane, which also increases TJ permeability (Oshima et al. 2007). Therefore, the suppression of oxidative stress or pro-inflammatory cytokines protects the intestinal barrier function.

Recent studies have reported the effects of nutrients on intestinal inflammation. Probiotics, polyphenols, and amino acids have been shown to reduce intestinal barrier dysfunction (Anderson et al. 2010; Beutheu et al. 2013; Carrasco-Pozo et al. 2013). For example, cysteine, an amino acid, increases the expression of TJ proteins and decreases the expression of pro-inflammatory cytokines, thus suppressing colitis-induced intestinal inflammation in piglets (Kim et al. 2009). Moreover, cysteine suppresses the NF-κB signaling pathway and activates the Nrf2 signaling pathway to trigger anti-inflammatory and antioxidative responses, alleviating intestinal barrier dysfunction (Song et al. 2016). Cysteine is used for the synthesis of glutathione (GSH), an antioxidant with anti-inflammatory effects (Diaz de Barboza et al. 2017). These studies suggest two possible mechanisms for the ameliorative effects of cysteine on the intestinal barrier dysfunction: the first mechanism involves the local effect of cysteine on the epithelial cells, and the other is by suppressing oxidative stress and/or inflammation systemically. Cysteine mainly exists in the cystine (Cys2) form because under normoxic conditions, cysteine is rapidly oxidized to Cys2 (Yin et al. 2016). Therefore, to clarify the effect of cysteine in the diet, it is necessary to identify whether Cys2 affects intestinal barrier dysfunction locally or systemically. Cys2 is known to inhibit the LPS-induced IL-6 production in monocytes (Tanaka et al. 2015) and enhance glutathione synthesis by incorporating into antigen-presenting cells (Rimaniol et al. 2001) in in vitro studies. These studies suggest that Cys2 locally suppresses inflammation and oxidative stress. However, the mechanism underlying the beneficial effect of Cys2 on intestinal barrier dysfunction systemically or locally is unclear.

Therefore, we hypothesized that Cys2 locally suppresses oxidative stress-induced intestinal barrier dysfunction. To test our hypothesis, we subjected the differentiated human colorectal adenocarcinoma (Caco-2) cell line, a model of the intestinal barrier with functional TJ complexes (Sambuy et al. 2005), to an H_2_O_2_ treatment and investigated the effects of cystine treatment on paracellular permeability, TJ protein expression, and intestinal inflammation.

## Materials and methods

### Reagents

Dulbecco’s modified Eagle’s medium (low glucose) with L-glutamine (DMEM), Dulbecco’s phosphate-buffered saline (DPBS), hydrogen peroxide, hydrochloric acid, and 100% w/v of trichloroacetic acid solution (TCA) were purchased from Wako Pure Chemical Industries (Osaka, Japan). Fetal bovine serum (FBS) was obtained from ICN Biomedicals (Costa Mesa, CA, USA). Minimum essential medium (MEM) with non-essential amino acids was obtained from Gibco™ (Co Dublin, Ireland). Penicillin–streptomycin was purchased from Nacalai Tesque (Kyoto, Japan). Trypsin–EDTA solution, L-cystine dihydrochloride (Cys2), and fluorescein isothiocyanate–dextran 4 kDa (FD4) were purchased from Sigma Chemical Company (St. Louis, MO, USA).

### Cell culture

Caco-2 cells were maintained in DMEM with 10% FBS, 1% penicillin–streptomycin, and 1% MEM at 37 °C and 5% CO_2_. Cells were seeded in 100-mm dishes, at a density of 0.6 × 10^5^ cells/mL/dish, and the medium was changed every 2–3 days, when cells were 80–90% confluent. For the measurement of cellular permeability, 0.2 × 10^5^ cells/well were seeded on 24-cell culture inserts (0.33 cm^2^, 0.4 µm pore size; Corning, Inc., Corning, NY), and differentiated confluent monolayers of Caco-2 cells, obtained after 14–18 days, were used in the transwell experiments. The amino acid and glucose composition of DMEM and MEM is shown in Table 1.

**Table 1:** Amino acid and glucose composition of DMEM and MEM

Amino acid and glucose	Concentration (mM)
l-Alanine	0.1
l-Asparagine	0.1
l-Aspartic acid	0.1
l-Arginine	0.4
l-Cystine	0.15
l-Glutamic acid	0.1
l-Glutamine	4
Glycine	0.5
l-Histidine	0.2
l-Isoleucine	0.8
l-Leucine	0.8
l-Lysine	0.8
l-Methionine	0.2
l-Phenylalanine	0.4
l-Proline	0.1
l-Serine	0.5
L-Threonine	0.8
l-Tryptophan	0.08
l-Tyrosine	0.27
l-Valine	0.8
d-Glucose	5.6

### Cys2 pretreatment and oxidative stress

For the measurement of cellular permeability, prior to oxidative stress, cells were pretreated with DMEM (control) or DMEM supplemented with different concentrations of Cys2 (0.1, 0.3, and 1 mM) in the apical compartments for 24 h and then incubated with 0.5 mM H_2_O_2_ for 1, 2, or 4 h. The protocol used to induce oxidative stress is as previously published (Iraha et al. 2013). Thereafter, transepithelial electric resistance (TEER) and paracellular permeability were measured, and cells were harvested for the estimation of mRNA expression. For the measurement of GSH and TJ proteins, Caco-2 cells were pretreated with DMEM (control) or DMEM supplemented with Cys2 (0.3 mM) for 24 h. For western blotting, Caco-2 cells were pretreated with DMEM supplemented with Cys2 (0.3 mM) and then stimulated with 0.5 mM H_2_O_2_ for 2 h. We selected the dose of Cys2 pretreatment based on the effect of Cys2 on H_2_O_2_-induced barrier dysfunction, cytotoxicity, and physiological level. Cys2 (0.3 mM) alleviated the H_2_O_2_-induced decrease in TEER and did not affect cell viability. A previous study has shown that after 200 mg/kg of Cys2 was orally administered to mice, approximately 0.2 mM Cys2 was transported into the plasma for 4 h, and levels in the plasma increased up to 2.7 times (Kurihara et al. 2007). Pretreatment was performed with 0.3 mM Cys2, which was twice the concentration of Cys2 in the basal medium (0.15 mM); therefore, we assumed that the physiological level of Cys2 was 0.3 mM and selected this as the concentration in our experiment.

### Measurement of TJ permeability

TEER, an indicator of TJ permeability to ionic solutes, and the paracellular flux of FD4, an indicator of TJ permeability to uncharged macromolecules, were assessed in monolayers of Caco-2 cells. TEER was measured using an EVOM^2^ meter (World Precision Instruments, FL, USA) after 1, 2, and 4 h of H_2_O_2_ stimulation. FD4 (1 mg/mL) was added to the apical side of the culture, at the time of H_2_O_2_ stimulation. Thereafter, 100 µL of the basolateral medium was transferred into 96-well plates after 1, 2, and 4 h of incubation with FD4 and H_2_O_2._ The fluorescence of the basolateral medium (excitation at 490 nm and emission at 520 nm) was measured using a SpectraMax^®^ i3x (Molecular Devices Japan, Tokyo, Japan). The results are represented as a percentage of the fluorescence of the non-stress group.

### Cell viability assay

The cytotoxicity of H_2_O_2_ and Cys2 to Caco-2 cells was evaluated by Cell Count Reagent SF (Nacalai Tesque, Kyoto, Japan) after incubation with different concentrations of H_2_O_2_ (0, 0.5, and 1 mM) for 2 h or pretreatment with DMEM supplemented with different concentrations of Cys2 (0, 0.1, 0.3, and 1 mM) for 24 h. The dehydrogenase reactions of NAD^+^ and NADH were measured for 1 h as the parameter for cell viability.

### RNA extraction and quantitative real-time polymerase chain reaction (RT-PCR)

Total RNA was extracted using the RNeasy mini kit™ (Qiagen, Hilden, Germany). The RNA quantification and purity were estimated by spectrophotometric readings of absorptions at 260 and 280 nm. The complementary DNA was synthesized using the Prime Script™ RT reagent Kit (Takara, Shiga, Japan). Gene expression of *IL-1β*, *TNF-α*, and heme oxygenase-1 (*HO-1*) was quantified using the SYBR Green analysis, on the Quant Studio™ 12 K Flex Real-Time PCR System (Applied Biosystems, Inc., Foster City, CA, USA). The relative mRNA levels were calculated using cycle threshold (Ct) values, normalized to the Ct values of 18S ribosomal RNA (18S rRNA). The following primers were used for the RT-PCR analysis:

*HO-1* forward primer, 5′-ATGAACTCCCTGGAGATGACTC-3′;

*HO-1* reverse primer, 5′- CCTTGGTGTCATGGGTCAG-3′;

*IL-1β* forward primer, 5′-CCAGGGACAGGATATGGAGCA-3′;

*IL-1β* reverse primer, 5′-TTCAACACGCAGGACAGGTACAG-3′;

*TNF-α* forward primer, 5′-CTGCCTGCTGCACTTTGGAG -3′;

*TNF-α* reverse primer, 5′- ACATGGGCTACAGGCTTGTCACT-3′;

18S rRNA, forward primer, 5′-CGCCGCTAGAGGTGAAATTC-3′;

and 18S rRNA, reverse primer, 5′-TTGGCAAATGCTTTCGCTC-3′.

### Western blotting analysis

TJ proteins were measured in whole cells, detergent-soluble fractions, and insoluble fractions to evaluate the content and localization of TJ proteins in CaCo-2. The detergent-insoluble fraction contained the actin cytoskeleton-associated proteins and the soluble fraction contained the intracellular proteins. Caco-2 cells pretreated with 0.3 mM Cys2 for 24 h and exposed to H_2_O_2_ for 2 h were lysed using 200 µL of buffer-CS (1% Triton X-100, 5 mM EGTA in 50 mM Tris containing protease and phosphatase inhibitor cocktails; Nacalai Tesque, Kyoto, Japan). Cell lysates were centrifuged at 15,600 × *g* for 10 min at 4 °C to precipitate the high-density actin-rich fraction. Pellets were resuspended in 100 µL of lysis buffer F (1% SDS, 1% Triton X-100, 1% sodium deoxycholate, 30 mM Tris, and the protease and phosphatase inhibitors cocktails described above, pH 7.4). For preparation of the whole Caco-2 cell extracts, 200 µL of lysis buffer F was used after washing cell monolayers with ice-cold PBS. Total protein concentration was assessed using a BCA protein assay kit (ThermoFisher, Waltham, MA, USA). Equal amounts of protein were boiled in 4 × NuPage LDS with dithiothreitol (ThermoFisher, Waltham, MA, USA). Boiled samples (20 µg whole cells, 10 µg soluble fractions, and 3.8 µg insoluble fractions) were separated by electrophoresis, using a 10% or 8–16% Criterion™ Tris–HCL Gel (Bio-Rad Laboratories Inc., Hercules, CA) in 10 × Tris/Glycine/SDS buffer (Bio-Rad Laboratories Inc., Hercules, CA, USA) and electro-transferred onto polyvinylidene difluoride membranes (Bio-Rad Laboratories Inc., Hercules, CA, USA) in a blotting buffer comprising 15% methanol and 10 × Tris/glycine buffer (Bio-Rad Laboratories Inc.). Membranes were blocked with StartingBlock™ (PBS) Blocking Buffer (ThermoFisher, Waltham, MA, USA) and incubated overnight at 4 °C with antibodies against ZO-1 (1:500, 61–7300; Invitrogen, Carlsbad, CA, USA), occludin (1:250, 40–4700; Invitrogen, Carlsbad, CA, USA), claudin-1 (1:250, 71–7800; Invitrogen, Carlsbad, CA, USA), and claudin-4 (1:500, 32–9400; Invitrogen, Carlsbad, CA, USA). Membranes were subsequently probed with anti-GAPDH antibody (1:2000, ab37168; Abcam, Cambridge, UK) to ensure equal loading. Membranes were washed four times for 5 min each with PBS solution and supplemented with 0.1% Tween-20 (PBST). Membranes were then incubated with peroxidase-IgG Fraction Monoclonal Mouse Anti-Rabbit IgG (1:10,000 dilution, 211–032-171; Jackson ImmunoResearch Inc., West Grove, PA) or Anti-mouse IgG, HRP-linked Antibody (1:1000 dilution, 7076; Cell Signaling Technology, Danvers, MA, USA) for 1 h at room temperature (20–25 ℃). Finally, blots were washed four times for 5 min each with PBST and incubated with ECL Prime western blotting Detection Reagent (Amersham Biosciences, Roosendaal, The Netherlands) for 5 min. Digital images were obtained, and signal intensities were quantified using the FUSION FX7 Imaging System (Witec AG, Sursee, Switzerland). Protein expression was normalized to GAPDH levels and expressed as mean fold change relative to the control group.

### Measurement of intracellular concentration of GSH, glutathione disulfide (GSSG), and Cys2

A total of 50 μL of cell suspension, treated with 10% trichloroacetic acid, was mixed thoroughly with an equal amount of dichloromethane and centrifuged at 14,000 × g for 2 min at 4 °C. The supernatant was collected and diluted 16-fold using the solvent of the mobile phase for liquid chromatography electron capture dissociation (LC-ECD) analysis. Chromatographic separation was carried out on an a ODS-3 (5.0 μm, 3 × 150 mm column; GL Sciences Inc., Tokyo, Japan) with an HPLC system composed of a GL-7410 pump, GL-7420 autosampler, GL-7430 oven, and ED703 pulse electrochemical detector (GL Sciences Inc., Tokyo, Japan). Briefly, 20 μL of the sample was injected in the HPLC column, with 50 mM NaH_2_PO_4_, 1 mM 1-octanesulfonic acid sodium salt, and 2.5% (v/v) acetonitrile solution (pH 2.7) as the mobile phase. The pump flow rate was initially set at 0.2 mL/min for 10 min, then at 0.8 mL/min for 20 min, and finally at 0.2 mL/min for 10 min. The column oven was maintained at 30 °C. The applied potential in the electrochemical detector was set at + 1800 mV. A calibration curve was constructed using a series of standard solutions with known concentrations of constituents. GSH, GSSG, and Cys2 concentrations in the sample were calculated by comparing the peak area of the chromatogram with that of the calibration curve. The concentration of Cys2, GSH, and GSSG was normalized to the protein content. Total protein concentration was assessed using a BCA protein assay kit (ThermoFisher, Waltham, MA, USA).

### Statistical analysis

Results are expressed as the mean ± standard error of mean (SEM). Comparisons between two groups were made using Student’s unpaired *t* tests. Results of paracellular permeability were analyzed using two-way ANOVA, followed by Dunnett’s multiple comparison test. For experiments involving more than three groups, Dunnett’s multiple comparison test was used. In addition, Spearman’s correlation test was used to analyze the relationship between gene expression of inflammatory cytokines and paracellular permeability. Data were analyzed using the GraphPad Prism 8 software (GraphPad Software Inc., San Diego, CA, USA). Results were considered statistically significant when *P* < 0.05.

## Results

### Effect of Cys2 on H_2_O_2_-induced barrier dysfunction in monolayers of Caco-2 cells

**Fig. 1:**
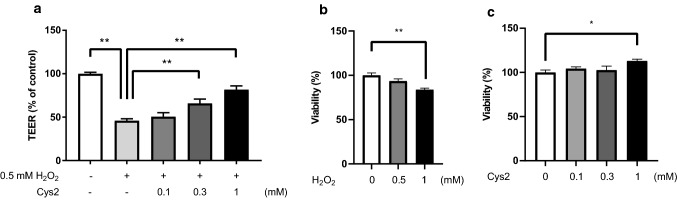
Cystine (Cys2) alleviates H_2_O_2_-induced intestinal barrier dysfunction. (**a**) Transepithelial electrical resistance (TEER) was measured across monolayers of Caco-2 cells incubated with 0.5 mM H_2_O_2_ for 2 h, with or without a Cys2 pretreatment of different concentrations (0.1, 0.3, and 1 mM) for 24 h. **P < 0.01 relative to TEER value of monolayers of Caco-2 cells treated with 0.5 mM H_2_O_2_. Values are represented as mean ± standard error of mean (*n* = 8–12). Cell viability was measured after with or without an H_2_O_2_ incubation of different concentrations (0.5, and 1 mM) for 2 h (**b**), and after with or without a Cys2 pretreatment of different concentrations (0.1, 0.3, and 1 mM) for 24 h (**c**). *P < 0.05 relative to cell viability of monolayers of Caco-2 cells incubated with DMEM. Values are represented as mean ± standard error of mean (*n* = 6)

To investigate the effect of Cys2 on H_2_O_2_-induced barrier dysfunction, the TEER of monolayers of Caco-2 cells was measured 2 h after the addition of 0.5 mM H_2_O_2_. H_2_O_2_ significantly decreased the TEER, and Cys2 pretreatment alleviated this H_2_O_2_-induced decrease in TEER in a dose-dependent manner (Fig. 1a,  *P*< 0.01). Incubation with 0.5 mM H_2_O_2_ did not change the cell viability, whereas incubation with 1 mM H_2_O_2_ significantly decreased the cell viability (Fig. 1b,  *P*< 0.05). Although 0.1 and 0.3 mM Cys2 pretreatment did not change the cell viability, 1 mM Cys2 pretreatment significantly increased the cell viability (Fig. 1c, *P* < 0.05).

### Effect of Cys2 on H_2_O_2_-induced changes in TJ permeability

**Fig. 2:**
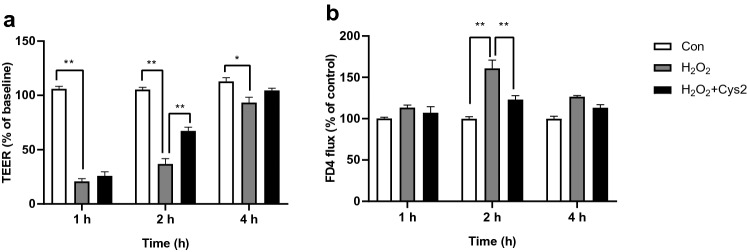
Cystine (Cys2) promoted recovery from H_2_O_2_-induced intestinal barrier dysfunction. **a** Transepithelial electrical resistance (TEER) was measured across monolayers of Caco-2 cells pretreated with 0.3 mM Cys2 for 24 h after 1, 2, and 4 h of incubation with 0.5 mM H_2_O_2_. Values are represented as mean ± SEM (*n* = 3–9). **b** Paracellular flux was measured across monolayers of Caco-2 cells pretreated with 0.3 mM Cys2 for 24 h after 1, 2, and 4 h of incubation with 0.5 mM H_2_O_2_. Control cells (Con) were monolayers pretreated with DMEM and not incubated with 0.5 mM H_2_O_2_. *P < 0.05, **P < 0.01 relative to TEER value of monolayers of Caco-2 cells treated with 0.5 mM H_2_O_2_. Values are represented as mean ± standard error of mean (*n* = 3–9)

We measured the TEER of cells incubated with 0.5 mM H_2_O_2_ for 1, 2, and 4 h to investigate the effect of Cys2 on TJ permeability. Although, TEER significantly decreased at 1 h (Fig. 2a,  *P*< 0.01), it recovered at 4 h. Cys2 pretreatment significantly reduced the decrease in TEER observed at 2 h of incubation with H_2_O_2_ (Fig. 2a,  *P*< 0.01). While FD4 flux increased at 2 h, Cys2 pretreatment significantly alleviated FD4 flux (Fig. 2b,  *P*< 0.01). These results indicate that Cys2 promotes recovery from oxidative stress-induced barrier dysfunction.

### Effect of Cys2 on the expression of TJ proteins

**Fig. 3:**
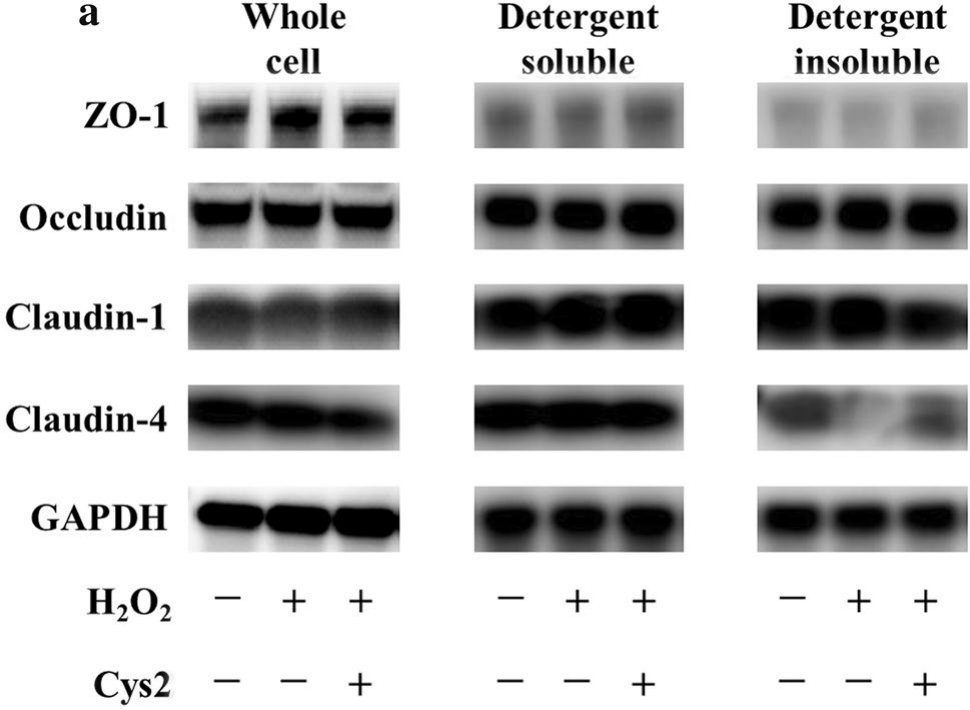

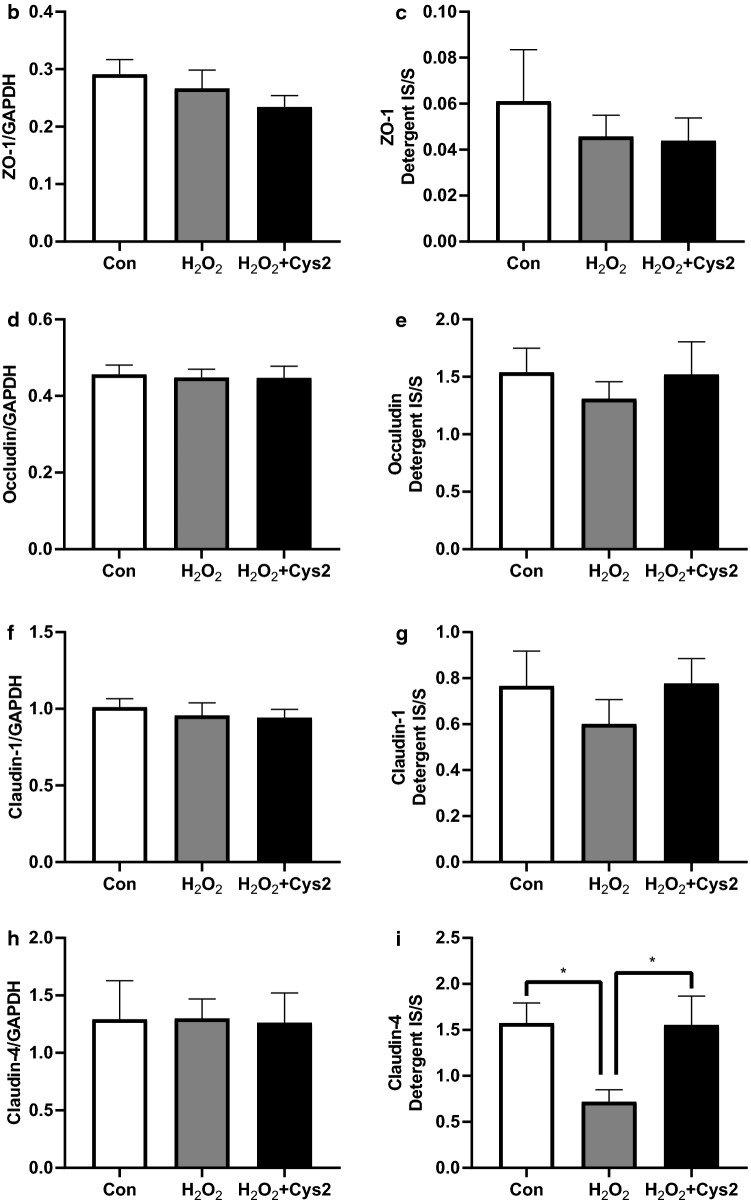
Cystine (Cys2) improved the decrease of the ratio of claudin-4 in the detergent-insoluble fractions (IS) to soluble fractions (S). Caco-2 cells were separated the detergent-insoluble fractions and soluble fractions after 2 h of incubation with 0.5 mM H_2_O_2_. **a** Whole cell extracts, detergent-soluble fractions and insoluble fractions of Caco-2 cells were immunoblotted for ZO-1, occludin, claudin-1, claudin-4, and GAPDH. The expression level of the TJ proteins zonula occludens-1 (ZO-1) (**b**), occludin (**c**), claudin-1 (**d**), and claudin-4 (**e**) in the whole cell extracts was measured and the ratio of TJ proteins in the detergent-insoluble fractions to soluble fractions was calculated. Control cells (Con) were monolayers pretreated with DMEM and not incubated with 0.5 mM H_2_O_2_. Protein expression was normalized to GAPDH levels and expressed as mean fold change relative to the Con group. Values are represented as mean ± SEM (*n* = 6)

To investigate the effect of Cys2 on the expression of TJ proteins, we measured the expression of ZO-1, occludin, and claudin-1 and -4 in Caco-2 cells incubated with H_2_O_2_ for 2 h by western blotting (Fig. 3a). There were no significant differences between the control and H_2_O_2_-treated groups in the expression of TJ proteins in the whole cells (Fig. 3b–e). The ratio of ZO-1, occludin, and claudin-1 in the detergent-insoluble fractions to soluble fractions were unchanged after stimulation with H_2_O_2_ (Fig. 3b–d), whereas the ratio of claudin-4 in the detergent-insoluble fractions to soluble fractions significantly decreased after stimulation with H_2_O_2_ (Fig. 3e,  *P*< 0.05). Further, Cys2 pretreatment significantly increased the ratio of claudin-4 in the detergent-insoluble fractions to soluble fractions (*P* < 0.05).

### Effect of Cys2 on oxidative stress

**Fig. 4:**
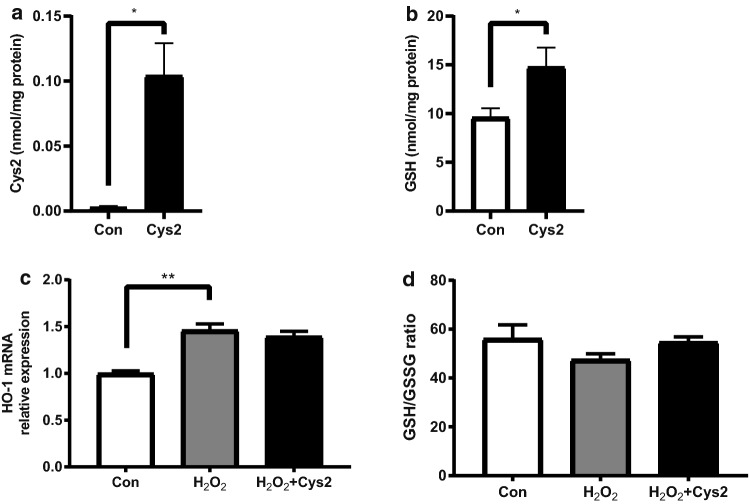
Cystine (Cys2) increased intracellular glutathione (GSH); however, it did not change the levels of oxidative stress indicators. The differentiated Caco-2 cells were treated with or without 0.3 mM Cys2 for 24 h. Intracellular levels of Cys2 (**a**) and GSH (**b**) were determined. The concentration of Cys2 and GSH was normalized to protein content. *P < 0.05. Values are represented as mean ± SEM (*n* = 3–6). **c** *HO-1* gene expression after 2 h of incubation with 0.5 mM H_2_O_2_. **P < 0.01 relative to expression values of cells treated 0.5 mM H_2_O_2_. Values are represented as mean ± SEM (*n* = 5–6). **d** The GSH/glutathione disulfide (GSSG) ratio after 2 h of incubation with 0.5 mM H_2_O_2_ was calculated from GSH and GSSG values. Values are represented as mean ± standard error of mean (*n* = 6). Control cells (Con) were monolayers pretreated with DMEM and not incubated with 0.5 mM H_2_O_2_

We measured the intercellular Cys2 and GSH levels to quantify the intercellular uptake of Cys2 and production of GSH after 24 h of incubation with 0.3 mM Cys2. Intercellular levels of Cys2 and GSH significantly increased in cells treated with 0.3 mM Cys2 compared to those in control cells (Fig. 4a, b,  *P*< 0.05). *HO-1* gene expression increased by 1.5 times in cells incubated with H_2_O_2_ for 2 h compared to that in control cells (Fig. 4c,  *P*< 0.01). *HO-1* gene expression was not significantly different between the H_2_O_2_ group and H_2_O_2_ + Cys2 group. The GSH-to-GSSG ratio was not significantly different between the H_2_O_2_ group and the other groups (Fig. 4d).

### Effect of Cys2 on H_2_O_2_-induced inflammation

**Fig. 5:**
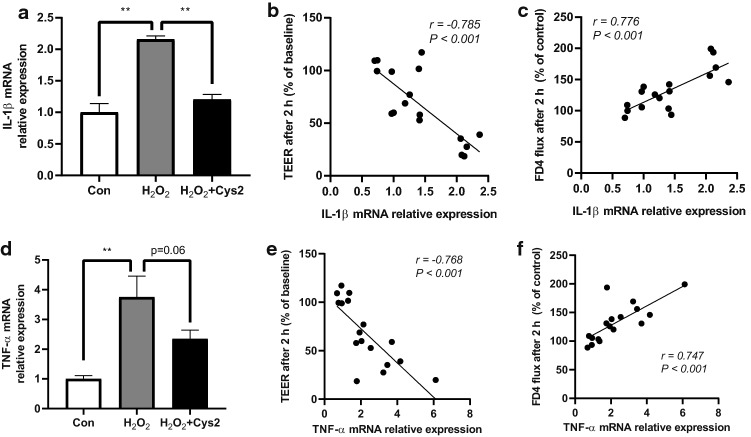
Cystine (Cys2) reduced H_2_O_2_-induced inflammation and intestinal barrier dysfunction. **a** *IL-1β* gene expression in control Caco-2 (Con) cells and Caco-2 cells incubated with H_2_O_2_, with or without a Cys2 pretreatment. **P < 0.01 relative to gene expression values of cells incubated with H_2_O_2_. Values are represented as mean ± standard error of mean (*n* = 5–6). The correlation of relative gene expression of *IL-1β* with (**b**) transepithelial electrical resistance (TEER) and (**c**) paracellular flux. **d** *TNF-α* gene expression in Con cells and Caco-2 cells incubated with H_2_O_2_, with or without Cys2 pretreatment. **P < 0.01 relative to H_2_O_2_ value. Values are represented as mean ± standard error mean (*n* = 5–6). The correlation of relative gene expression of *TNF-α* with (**e**) TEER and (**f**) paracellular flux

We found that the gene expression of *IL-1β* was significantly increased (Fig. 5a,  *P*< 0.01) in cells incubated with 0.5 mM H_2_O_2_ for 2 h. On the contrary, the gene expression of *IL-1β* was significantly more suppressed in Cys2-pretreated cells incubated with H_2_O_2_ than that in cells incubated with H_2_O_2_ (*P* < 0.01). In addition, *IL-1β* gene expression was significantly correlated with TEER (*r* = − 0.785, *P* < 0.001, Fig. 5b) and FD4 flux (*r* = 0.776, *P* < 0.001, Fig. 5c). *TNF-α* gene expression was significantly increased in cells incubated with 0.5 mM H_2_O_2_ for 2 h (Fig. 5d,  *P*< 0.01) and tended to be suppressed in Cys2-pretreated cells incubated with H_2_O_2_ compared to that in cells incubated with H_2_O_2_ (*P* < 0.01). In addition, *TNF-α* gene expression was significantly correlated with TEER (*r* = − 0.768, *P* < 0.001, Fig. 5e) and FD4 flux (*r* = 0.747, *P* < 0.001, Fig. 5f).

## Discussion

In this study, we established an oxidative stress-induced model using H_2_O_2_ to identify whether Cys2 locally improved intestinal barrier dysfunction. We found that TEER of Caco-2 cells significantly decreased after 1 h incubation and recovered after 4 h incubation with H_2_O_2_. Furthermore, FD4 permeability significantly increased after 2 h incubation and recovered after 4 h incubation with H_2_O_2_. Contrary to our results, a previous report has shown that TEER significantly decreased after exposure to 0.5 mM H_2_O_2_ for 2 h and continued to decrease for 6 h after H_2_O_2_ exposure (Iraha et al. 2013). In this study, the expression of ZO-1, occludin, and claudin-1 and -4 in whole cells was not altered by oxidative stress. The difference in results between the previous study and ours might derive from the initial condition of the TJ structure. In the previous study, TEER was estimated to be 600 Ω/cm^2^ whereas, in our study, TEER was estimated to be 1500 Ω/cm^2^ before exposure to oxidative stress. Therefore, the TJ structure was more robust in our study than in the previous study, and the oxidative stress induced by 0.5 mM H_2_O_2_ was not sufficient to reduce the expression of TJ proteins. Moreover, the cell viability did not change after exposure to 0.5 mM H_2_O_2_ for 2 h. Thus, these results indicate that our experimental model likely mimics temporary oxidative stress-induced intestinal barrier dysfunction, such as the exercise-induced increase in gastrointestinal permeability, instead of chronically impaired barrier function that would lead to a decreased expression of TJ proteins, such as during IBD in humans (Michielan and D'Inca 2015; van Wijck et al. 2011).

Oxidative stress is known to increase the expression of pro-inflammatory cytokines and decrease that of TJ proteins, resulting in intestinal barrier dysfunction (Liu et al. 2019). Another study has shown that oxidative stress does not change the expression levels of TJ proteins in whole cells but changes the localization of TJ proteins from the apical membrane to the lateral membrane, inducing intestinal barrier dysfunction (Oshima et al. 2007). In this study, the expression levels of TJ proteins in whole cells did not change; however, the ratio of claudin-4 protein in the detergent-insoluble fractions to soluble fractions significantly decreased. Pro-inflammatory cytokines change the myosin binding structure via myosin light chain kinase-mediated phosphorylation, leading to the internalization of TJ proteins by endocytosis and an increase in paracellular permeability (Du et al. 2016). When the internalized TJ proteins return to the cell membrane, paracellular permeability is restored to the basal level (Jin and Blikslager 2016; Marchiando et al. 2010). Therefore, in our study, the oxidative stress-induced intestinal barrier dysfunction may have been caused by the recycling of TJ proteins in response to inflammation.

Pretreatment with Cys2 for 24 h did not suppress the oxidative stress-induced increase in TEER of cells incubated with H_2_O_2_ for 1 h. However, after 2 h of incubation with H_2_O_2_, the TEER was significantly increased in Cys2-pretreated cells compared to that in cells that had not been pretreated; moreover, the H_2_O_2_-mediated increase in FD4 flux was suppressed by Cys2 pretreatment. These results suggested that Cys2 promoted the recovery of oxidative stress-induced intestinal barrier dysfunction.

Previous reports suggested that Cys2 is taken up by cells via a cystine/glutamate transporter on the plasma membrane and metabolized to cysteine for GSH synthesis (Rimaniol et al. 2001). In our study, Cys2 pretreatment significantly increased intracellular Cys2 and GSH concentrations. γ-Glutamyl cysteine synthetase is the rate-limiting enzyme for GSH synthesis, and sulfur amino acid intake is rate-limiting for GSH synthesis (Grimble 2006). Therefore, we speculated that Cys2 supplementation mainly contributed to promote GSH synthesis. Intestinal barrier dysfunction is presumed to have been reduced due to the suppression of oxidative stress by the increase in GSH. However, the GSH/GSSG ratio did not show any change after 2 h of oxidative stress; furthermore, Cys2 pretreatment did not affect the GSH/GSSG ratio in cells exposed to oxidative stress for 2 h. In addition, we tested *HO-1* gene expression and cell viability as markers of oxidative stress, as gene expression of *HO-1* is known to be upregulated in response to oxidative stress (Ryter and Choi 2009). In our study, a slight increase in *HO-1* gene expression was noted after 2 h of oxidative stress compared to that in the control group, indicating that oxidative stress was slightly induced in Caco-2 cells, and Cys2 pretreatment had no effect on the *HO-1* gene expression. A recent study has shown that a high dose of H_2_O_2_ (more than 1 mM) decreases the cell viability in Caco-2 cells (Yang et al. 2019), while incubation with 0.5 mM H_2_O_2_ does not change the cell viability. Therefore, the oxidative stress induced by 0.5 mM H_2_O_2_ in our study was not sufficient to decrease the cell viability. These results indicate that Cys2 is taken up into the intestinal cells, wherein it promotes GSH synthesis, before H_2_O_2_ exposure; however, we did not observe any antioxidative response due to weak oxidative stress induced by H_2_O_2_.

As described above, the impaired barrier function in our study is thought to be due to intestinal inflammation. It is known that the opening of TJ leads to an influx of LPS into the cell, resulting in the expression of pro-inflammatory cytokines (Raetz and Whitfield 2002). Previous reports have shown that Caco-2 cells cultured with N-acetylcysteine, a precursor of GSH, suppress the MAPK signaling pathway, which, in turn, suppress LPS-induced production of TNF-α and IL-6 (Haddad 2002). As Cys2 promotes GSH synthesis, Cys2 is believed to suppress inflammation through a similar pathway. Another report has shown that oxidative stress changes the localization of claudin-4 by activating the p38 MAPK, and the p38 MAPK inactivator Wip1 significantly attenuates H_2_O_2_-induced permeability and claudin-4 delocalization (Oshima et al. 2007). In this study, we found that the expression of *IL-1β* and *TNF-α* was both upregulated by oxidative stress and downregulated by Cys2 pretreatment. In addition, we found that the gene expression of inflammatory cytokines was negatively correlated to TEER and positively correlated to FD4 flux. Furthermore, Cys2 attenuated the H_2_O_2_-induced claudin-4 delocalization. Collectively, these results suggest that Cys2 may alleviate barrier dysfunction by suppressing the production of pro-inflammatory cytokines, possibly via the suppression of MAPK signaling. Further studies warrant detailed mechanisms of Cys2-mediated improvement of barrier function. 

Intestinal barrier function can decrease either chronically or transiently. For example, IBD- or CRC-induced decreases in the intestinal barrier function are usually chronic (Stidham and Higgins 2018), whereas the changes induced by high-intensity exercises—due to the exercise-induced oxidative stress in the intestines—are mostly transient (Lambert 2009), as the barrier function will recover following the cessation of the exercise. A previous study on humans reported increased gastrointestinal permeability after 60 min of high-intensity cycling exercise, which recovered 2 h after the exercise (van Wijck et al. 2011). In this study, H_2_O_2_ treatment induced a transient decrease in the intestinal barrier function, which recovered 4 h after the treatment. However, in a previous report (Iraha et al. 2013), the decrease in the intestinal barrier function persisted for 6 h after the H_2_O_2_ treatment. Therefore, the current model is thought to mimic the transient intestinal barrier dysfunction, such as the exercise-induced barrier dysfunction, and not those induced by chronic gastrointestinal diseases, such as IBD (Michielan and D'Inca 2015; van Wijck et al. 2011). Post-exercise barrier dysfunction is one of the major challenges for athletes, leading to fatigue and reduced performance (Clark and Mach 2016). Recently, nutritional supplements such as curcumin and probiotics have been shown to improve chronic barrier dysfunction in vitro under high-stress conditions (Seth et al. 2008; Wang et al. 2012). However, only a few dietary supplements are known to improve acute stress-induced transient intestinal barrier dysfunction. In our study, Cys2 could ameliorate the transient intestinal barrier dysfunction, suggesting that when taken prior to performing high-intensity exercises, Cys2 may improve athlete performance by reducing post-exercise barrier dysfunction.

We have shown Cys2 to locally ameliorate oxidative stress-induced barrier dysfunction in vitro. However, whether Cys2 secondarily improves intestinal barrier dysfunction by suppressing oxidative stress and/or systemic inflammation, as well as the local effect of Cys2 found in the current study, remains unclear. Thus, future studies are required to investigate the systemic effect of Cys2 ingestion on intestinal function. Our results have shown that the oxidative stress model in this study may induce intestinal barrier dysfunction through a different mechanism than the chronic oxidative stress model; however, the details of the mechanism are yet unclear. Thus, further studies are warranted to determine whether TJ protein translocation is induced by oxidative stress and increases TJ permeability.

In conclusion, we induced temporary intestinal barrier dysfunction in Caco-2 cells by the addition of a low dose of H_2_O_2_ into the culture medium. We revealed that Cys2 locally reduced oxidative stress-induced barrier dysfunction, possibly due to the change of TJ localization from an apical membrane to a lateral membrane. Furthermore, Cys2 increased intracellular GSH levels and suppressed oxidative stress-induced expression of pro-inflammatory cytokines. Therefore, Cys2 locally improved the intestinal barrier function by suppressing inflammatory responses.

## Data Availability

Data generated or analyzed during this study are included in this published article.
